# IL-6 trans-signaling in cystic fibrosis bronchial cells potentiates TNF-α-driven ICAM-1 expression

**DOI:** 10.3389/fcell.2025.1566482

**Published:** 2025-09-23

**Authors:** John Lin, Lyvia Fourcade, Lucie Roussel, Matthew Marabella, Julie Bérubé, Dao Nguyen, Simon Rousseau

**Affiliations:** The Meakins-Christie Laboratories at the Research Institute of the McGill University Health Centre and Department of Medicine, McGill University, Montreal, QC, Canada

**Keywords:** *Pseudomonas aeruginosa*, cystic fibrosis transmembrane regulator, cystic fibrosis, pulmonary exacerbations, inflammation, IL-6 trans-signaling, ICAM-1

## Abstract

**Introduction:**

*Pseudomonas aeruginosa* is gram-negative *bacillus* that causes chronic airway infections, leading to severe pulmonary inflammation in cystic fibrosis. This bacterial infection is frequently associated with a massive recruitment of neutrophils and an abnormal increase in production of inflammatory cytokines. Among these cytokines, interleukin (IL)-6 has both anti- and pro-inflammatory properties able to signal through classic and trans-signaling pathways, respectively. Furthermore, IL-6 is known to be upregulated in CFTR-deficient bronchial cell lines in the presence of *Pseudomonas aeruginosa*-derived filtrates and in Pulmonary Exacerbations (PEx). In this study, we aimed to determine whether IL-6 trans-signaling could contribute to neutrophilic inflammation leading to lung tissue damage during PEx of people with CF (pwCF).

**Methods:**

sIL-6Ra expression was measured by ELISA in plasma samples from pwCF at baseline and during exacerbations. IL-6 signalling was investigate in CF and non-CF cell lines using immunoblotting of STAT3 phosphorylation. ICAM-1 cell surface expression was determined using flow cytometry.

**Results:**

We show that pwCF had higher sIL-6Rα levels in their plasma during PEx, suggestive of IL-6 trans-signaling. Furthermore, we show that a CF bronchial cell line is hyper-responsive to both classic and trans-signaling, with the higher levels of activation occurring during trans-signaling when compared to two non-CF cell lines.

**Discussion:**

Our data unveiled that ICAM-1, which promotes neutrophil adhesion, is upregulated by the combination of TNF-α and IL-6 signaling in CF bronchial cells. Interestingly, soluble IL-6R (sIL-6Rα) protects IL-6 from degradation by bacterial proteases. Therefore, we suggest that strategies which target IL-6 trans-signaling may alleviate ICAM-1 mediated neutrophil adhesion and reduce subsequent lung damage in PEx.

## 1 Introduction

In cystic fibrosis (CF) lung disease, mutations in the cystic fibrosis transmembrane conductance regulator (CFTR) gene and chronic bacterial infections lead to a sustained pro-inflammatory state, resulting in excessive neutrophil recruitment to the airways (Castellani and Assael, 2017; Margaroli and Tirouvanziam, 2016; Hidalgo et al., 2022). The sustained pro-inflammatory state is the result of CFTR loss of function as inducible expression of CFTR attenuates pro-inflammatory cytokine secretion in human respiratory epithelial cells (Cabrini et al., 2020; Castaldo et al., 2020; Veit et al., 2012). Moreover, the loss of CFTR function leads to MAPK hyperactivation in airway epithelial cells exposed to bacterial ligands, resulting in increased expression of pro-inflammatory cytokines, such as the neutrophil chemoattractant IL-8 and the pleiotropic cytokine IL-6 (LaFayette et al., 2015; Bérubé et al., 2010).

IL-6 is a multifunctional cytokine that is involved in the initiation and resolution of inflammation (Tanaka et al., 2014). Over the years, this duality has led to conflicting reports regarding whether IL-6 is a friend or a foe in chronic or autoimmune diseases. A reason that may explain the ambiguity of IL-6 functions is the complex signaling network that mediates its activities. Two types of signaling mechanisms have been described: classic signaling and trans-signaling (Uciechowski and Dempke, 2020). In “*classic signaling*,” IL-6 binds to a complex formed by the transmembrane receptor IL-6Rα and the common cytokine receptor gp130. Although gp130 is expressed ubiquitously, membrane IL-6Rα has a more restricted expression pattern (Calabrese and Rose-John, 2014). Classic IL-6 signaling has been demonstrated to be essential for host defense against pathogens in multiple mouse models (Calabrese and Rose-John, 2014). Moreover, classic IL-6 signaling is important for ciliated epithelial cell differentiation, a function essential for epithelium repair and beneficial in CF lung disease (Tadokoro et al., 2014). As such, it is usually regarded as the beneficial arm of IL-6 action. Alternatively, in trans-signaling, the soluble form of IL-6Rα (sIL-6Rα) can bind IL-6 and initiate signaling via the ubiquitously expressed gp130 (Tadokoro et al., 2014). This occurs when the membrane-bound IL-6Rα is shed from cells such as neutrophils via the action of the protease ADAM17 (Marin et al., 2002; Yan et al., 2016). Interestingly, the signals leading to IL-6Rα shedding from neutrophils include CXC chemokines like IL-8 abundantly found in the lungs of people with CF (pwCF) (Marin et al., 2002). Trans-signaling is strongly associated with pro-inflammatory states (Rose-John, 2012) and has, therefore, been proposed as a potential target to reduce damaging inflammation in chronic inflammatory diseases (Calabrese and Rose-John, 2014).

Pulmonary exacerbations (PEx) cause significant morbidity and severely impact disease progression in CF (Sanders et al., 2010). The frequency of PEx in CF lung disease is associated with greater lung function decline and shorter time to transplantation (de Boer et al., 2011). Identifying the key factors responsible for increased tissue inflammation leading to lung function decline is essential to prevent the irreversible loss of pulmonary functions resulting from exacerbations. The hypothesis investigated in this research paper is that IL-6 trans-signaling occurs during PEx in the lungs of pwCF and could be an important contributing factor to lung inflammation and tissue destruction.

## 2 Materials and methods

### 2.1 Materials

All the chemicals were purchased from Fisher Scientific (Fair Lawn, NJ, United States). Antibodies used for immunoblotting and flow cytometry were purchased from Cell Signaling Technology (Danvers, MA, United States) and R&D Systems (Minneapolis, MN, United States), respectively. IL-6, sIL-6Rα, and TNF-α recombinant proteins were purchased from PeproTech (Cranbury, NJ, United States).

### 2.2 Clinical samples

Plasma blood samples were obtained from a cohort of previously described patients (Sanders et al., 2010; Wojewodka et al., 2014; Mizutani et al., 2017).

### 2.3 *Pseudomonas aeruginosa* diffusible material preparation

PA14, a highly virulent reference strain of *Pseudomonas aeruginosa* derived from a clinical isolate (He et al., 2004; Wang et al., 2010), was grown in LB (Luria broth) (Essar et al., 1990; Palmer et al., 2007). *P. aeruginosa* diffusible material was prepared and used as described previously (Beaudoin et al., 2013).

### 2.4 Epithelial cell culture

The BEAS-2B airway epithelial cells were identified as a non-CF bronchial epithelial cell model and were cultured in a serum-based medium (DMEM), as previously described (Bérubé et al., 2010). The UNC CF2 (CF2) airway epithelial cell line, which carries the most common mutation found in CF—the deletion of Phe508—was kindly provided by Dr. Scott Randell (The University of North Carolina at Chapel Hill, NC, United States). CF2 cells were cultured in a serum-free medium, as defined by Fulcher et al. (2009). The UNC N3T (N3) airway epithelial cell line was chosen as an additional non-CF bronchial epithelial cell model and was also kindly provided by Dr. Scott Randell. N3 cells were cultured in the serum-free medium, as described by Fulcher et al. (2009). To enhance cell adherence, cells were seeded onto a PureCol pre-coated plate (Advanced BioMatrix, San Diego, CA, United States).

### 2.5 Determination of sIL-6Rα concentration in plasma from CF patients

sIL-6Rα levels were determined using a commercial ELISA kit purchased from R&D Systems (Minneapolis, MN, United States) according to the manufacturer’s protocol.

### 2.6 Total protein extraction and immunoblotting

BEAS-2B, CF2, and N3 cell lines were each stimulated with IL-6 and sIL-6Rα for 30 min before being placed on ice to arrest cell activity. Cells were washed with cold PBS and lysed in a buffer (50 mM Tris–HCl, pH 7.5, 1 mM EGTA, 1 mM EDTA, 1% (v/v) Triton X-100, 1 mM sodium orthovanadate, 5 mM sodium pyrophosphate, 50 mM sodium fluoride, 0.27 M sucrose, and 5 mM sodium pyrophosphate) and protease inhibitor cocktail (Thermo Fisher Scientific, United States) for 10 min on ice. Supernatants were recovered by centrifugation at 12,000 x g at 4 °C for 5 min. Protein quantification was carried out using the Bio-Rad Protein Assay Dye Reagent (Bio-Rad, CA, United States). Samples were boiled in loading buffer (final concentration: 60 mM Tris–HCl, pH 6.8, 2% (w/v) SDS, and 10% (v/v) glycerol) supplemented with 10% (v/v) TCEP (Thermo Fisher Scientific) at 95 °C for 5 min and then loaded onto 12.5% NEXT GEL polyacrylamide gels (VWR, United States). Gels were run for 10 min at 70 V, followed by 120 min at 125 V, using the Mini-PROTEAN Tetra Vertical Electrophoresis Cell (Bio-Rad, United States) and wet-transferred at 100 V for 35 min at 4 °C in the transfer buffer onto 0.45 μm nitrocellulose membranes (Bio-Rad, CA, United States) using a Criterion Blotter wet-transfer apparatus (Bio-Rad, CA). Membranes were blocked in 5% milk/TBS-Tween solution for 1 h at ambient temperature before immunoblotting. Primary antibodies were used in the following dilutions: monoclonal rabbit anti-phospho-STAT-3 (Tyr705) (Cell Signaling, 9145), 1:1,000; monoclonal rabbit total anti-STAT-3 (D3Z2G) (Cell Signaling, 9132), 1:1,000; and monoclonal rabbit anti-IL-6 (D3K2N) (Cell Signaling, 12153), 1:1,000; these antibodies were diluted in 1% BSA/TBS-Tween and incubated at ambient temperature for 2 h. Secondary antibodies were used in the following dilutions: goat anti-rabbit IgG DyLight™ 800 and goat anti-mouse IgG DyLight™ 680, 1:15,000, in 1% milk/TBS-Tween at ambient temperature for 1 h. After incubation with antibodies, the membranes were washed three times with PBS-Tween. Quantitative analysis of the signals from each antibody was performed using the Li-COR Odyssey Infrared Imaging System.

### 2.7 Cell lysis, RNA extraction, and real-time PCR

BEAS-2B, CF2, and N3 cell lines were each stimulated with IL-6, sIL-6Rα, and TNF-α for 1 h before being placed on ice to arrest cell activity. Cell lysis, RNA extraction, and RT-PCR techniques were performed as previously described (Bérubé et al., 2010).

### 2.8 Flow cytometry analyses

Cell lines were each stimulated with IL-6, sIL-6Rα, and TNF-α for 24 h before being trypsinized. Cells were washed and processed for flow cytometry analysis. Cells were stained with ICAM-1/CD54 fluorescein-conjugated antibody (R&D Systems, United States) at 1:100 for 30 min at ambient temperature. Cells were washed and then fixed with 0.1% (w/v) paraformaldehyde at 4 °C for 10 min. Data acquisition was performed using BD FACSCanto™ II (BD Biosciences), and analysis was carried out using FlowJo 7.6.3 software (Tree Star, Ashland, OR, United States), where geometric mean and median fluorescence intensities were calculated.

### 2.9 Statistical analysis

Data from each condition were compared separately with the untreated conditions. The D’Agostino–Pearson normality test was used to determine whether the values were sampled from a Gaussian distribution. The statistical significance of differences between groups was determined using the Kruskal–Wallis test, followed by Dunn’s multiple comparisons post-test. Analyses were performed using GraphPad Prism 5.00 (GraphPad Software, CA, United States). *p*-values <0.05 were considered significant.

## 3 Results

### 3.1 Evidence for IL-6 trans-signaling during pulmonary exacerbations in cystic fibrosis

**FIGURE 1:**
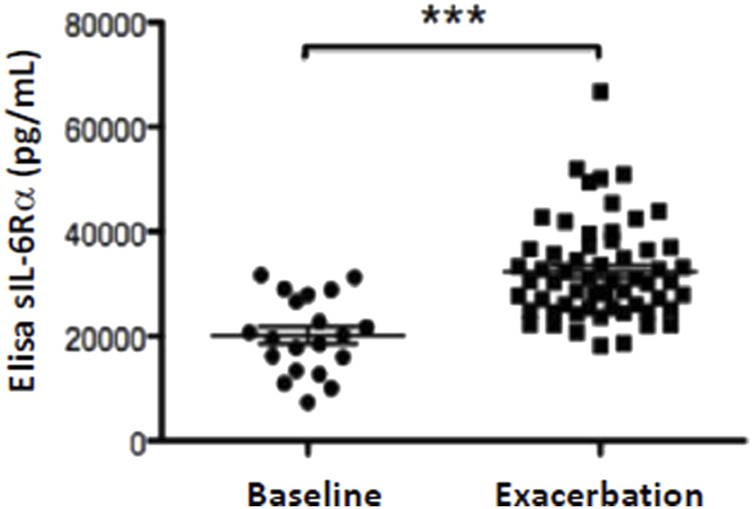
Levels of sIL-6Rα are increased during PEx. Concentrations of sIL-6Rα (pg/mL) in the blood plasma in pwCF experiencing PEx compared to baseline levels. Data are presented as the mean ± SD (or SEM) and were compared using unpaired t-test. Significance levels are shown as *** (*p* < 0.001). sIL-6Rα, soluble form of interleukin-6 receptor alpha; PEx, pulmonary exacerbation; CF, cystic fibrosis.

In a previously described cohort of pwCF (Wojewodka et al., 2014; Mizutani et al., 2017; Ahlgren et al., 2015), the plasma levels of sIL-6Rα were found to be significantly higher during PEx (Figure 1). The majority, but not all, of these pwCF showed positive cultures for *P. aeruginosa* (Wojewodka et al., 2014; Mizutani et al., 2017; Ahlgren et al., 2015). Considering previously reported data on higher levels of IL-6 during PEx determined in the same cohort of patients (Wojewodka et al., 2014), these results suggest that IL-6 trans-signaling may occur during PEx in the lungs of pwCF.

### 3.2 IL-6 classic and trans-signaling are higher in a CF bronchial cell line

**FIGURE 2:**
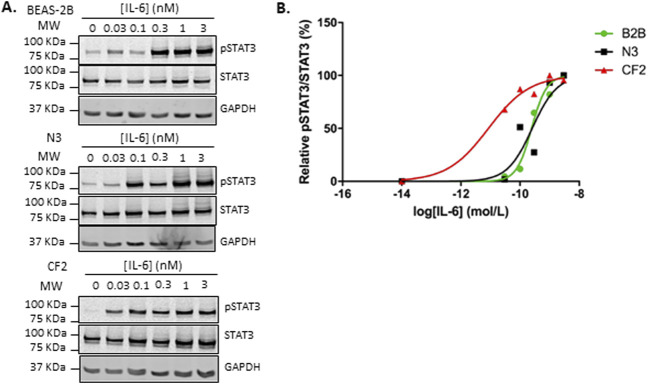
The CF2 bronchial epithelial cell line is more responsive to IL-6 signaling than the two non-CF bronchial epithelial cell lines. **(A)** Immunoblots showing phosphorylated STAT3 (pSTAT3), STAT3, and GAPDH expression levels in BEAS-2B (top panel), N3 (middle panel), and CF2 (bottom panel) cell lines stimulated with different concentrations of IL-6 (0 nM–3 nM) for 30 min. GAPDH was used as a loading control. **(B)** Quantification of STAT3 phosphorylation levels (pSTAT3/STAT3) expressed as the % of maximal phosphorylation in the immunoblots shown in **(A)** using a Li-COR Odyssey Imaging System. BEAS-2B, non-CF bronchial epithelial cells; N3, non-CF bronchial epithelial cells counterpart; CF2, CF bronchial epithelial cells; IL-6, interleukin-6; STAT3, signal transducer and activator of transcription 3.

To address the potential contribution of IL-6 trans-signaling to inflammation in the context of CF, the sensitivity of an immortalized CF bronchial cell line (CF2, isolated from a CFTR p.Phe508del homozygous person) was compared with a wild-type counterpart (N3). In contrast to virally transformed cell lines (such as BEAS-2B), these two cell lines were created using a protocol designed to better maintain normal cell structure and function (Fulcher et al., 2009). The IL-6 signaling network was investigated by quantifying the phosphorylation levels of STAT-3, a transcription factor that is well-known to be activated by both classic and trans-signaling. When exposed to increasing concentrations of IL-6, bronchial cells from a pwCF (CF2) showed a marked increase in sensitivity, especially at lower concentrations, compared to a non-CF counterpart (N3) or the non-CF bronchial epithelial cell line BEAS-2B (Figures 2A, B). This shows that bronchial epithelial cells can mediate IL-6 classic signaling.

**FIGURE 3:**
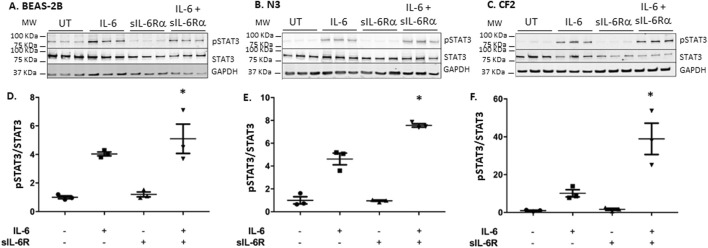
IL-6 trans-signaling occurs in bronchial epithelial cells. BEAS-2B, N3, and CF2 were stimulated in absence or presence of IL-6 and/or sIL-6Rα at 20 ng/mL each for 30 min **(A–C)** Cell lysates were immunoblotted, as shown in Figure 2. **(D–F)** Quantification of STAT3 phosphorylation levels was performed as shown in Figure 2, except that it is expressed as the fold change over untreated levels (the y-scale magnitude varies from one graph to the other). Data are representative of three different experiments. Data are presented as the mean ± SEM and were performed using the Kruskal–Wallis non-parametric test, followed by Dunn’s multiple comparisons post-test. Significance levels are shown as *(p < 0.05 compared to the untreated samples). BEAS-2B, non-CF bronchial epithelial cells; N3, non-CF bronchial epithelial cells counterpart; CF2, CF bronchial epithelial cells; STAT3, signal transducer and activator of transcription 3; sIL-6Rα, soluble interleukin-6 receptor alpha; IL-6, interleukin-6.

To determine whether this holds true for trans-signaling, the experiment was carried out in the absence or presence of sIL-6Rα added at an equimolar concentration to IL-6. In both the N3 and CF2 bronchial cells, adding sIL-6Rα in addition to IL-6 led to >2 times higher levels of STAT-3 phosphorylation than when exposing the cells to IL-6 alone (Figures 3A–F). When comparing CF2 to N3 cells, a net 5-fold increase in STAT-3 phosphorylation (non-CF = 8 times vs. CF = 40 times) was observed in CF2 cells. These results show that the CF2 bronchial cell line is hyper-responsive to both classic and trans-signaling, with the higher levels of activation occurring during trans-signaling.

### 3.3 IL-6 signaling increases TNF-α-driven ICAM-1 expression in CF airway epithelial cells

**FIGURE 4:**
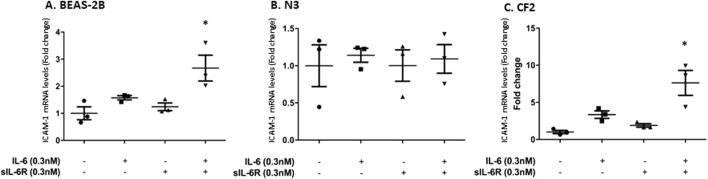
IL-6 signaling increases ICAM-1 mRNA expression in BEAS-2B and CF2 but not N3 cell lines. Cell lines were stimulated in absence or presence of IL-6 and/or sIL-6Rα at 0.3 nM each for 1 h. Expression level (fold change compared to the untreated cells) of ICAM-1 mRNA in **(A)** BEAS-2B, **(B)** N3, and **(C)** CF2 cell lines after treatment were assessed by qPCR. The y-scale magnitude varies from one graph to the other. Data are representative of three different experiments. Data are presented as the mean ± SEM and were performed using the Kruskal–Wallis non-parametric test, followed by Dunn’s multiple comparisons post-test. Significance levels are shown as *(p < 0.05 compared to the untreated samples). BEAS-2B, non-CF bronchial epithelial cells; N3, non-CF bronchial epithelial cells counterpart; CF2, CF bronchial epithelial cells; ICAM, inter-cellular adhesion molecule; sIL-6Rα, soluble interleukin-6 receptor alpha; IL-6, interleukin-6.

**FIGURE 5:**
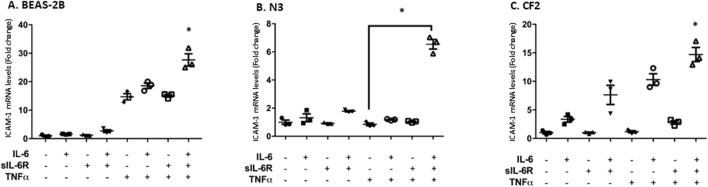
TNF-α and IL-6 co-stimulation increases ICAM-1 mRNA expression level to a greater level than the agonists separately. Each cell line was stimulated in absence or presence of IL-6, sIL-6Rα, and/or TNF-α at 20 ng/mL for 1 h. Fold change in the expression level of ICAM-1 mRNA in BEAS-2B **(A)**, N3 **(B)**, and CF2 **(C)** cell lines after treatment was assessed using qPCR. The y-scale magnitude varies from one graph to the other. Data are representative of three different experiments. Data are presented as the mean ± SEM and were performed using the Kruskal–Wallis non-parametric test, followed by Dunn’s multiple comparisons post-test. Significance levels are shown as *(p < 0.05 compared to the untreated samples). BEAS-2B, non-CF bronchial epithelial cells; N3, non-CF bronchial epithelial cells counterpart; CF2, CF bronchial epithelial cells; ICAM, inter-cellular adhesion molecule; TNF-α, tumor necrosis factor alpha; sIL-6Rα, soluble interleukin-6 receptor alpha; IL-6, interleukin-6.

The next question was to assess the impact of trans-signaling on downstream mediators of inflammation. Cytokines secreted by immune cells, such as TNF-α, upregulate intercellular adhesion molecule-1 (ICAM-1), a cell surface adhesion molecule that can bind leukocytes via LFA-1 (Chan et al., 2008). ICAM-1 is also a STAT-3 target gene (Rakemann et al., 1999; Huang et al., 2014; Takeda et al., 1997). Stimulating bronchial cells with IL-6 alone or in combination with sIL-6Rα led to a modest increase in ICAM-1 mRNA expression determined by qPCR that was the highest in CF2 cells, which is in accordance with the higher phosphorylation of STAT-3 shown in the previous section (Figures 4A, C). No changes were observed in the N3 cell line. In the N3 and CF2 cell lines, stimulation with TNF-α alone failed to increase ICAM-1 mRNA expression (Figures 5B, C). However, synergistic activation of ICAM-1 mRNA was observed when TNF-α stimulation was combined with IL-6 trans-signaling (Figures 5A–C). In accordance with prior results, the highest level of ICAM-1 expression was achieved when TNF-α was combined with IL-6 trans-signaling in CF2.

**FIGURE 6:**
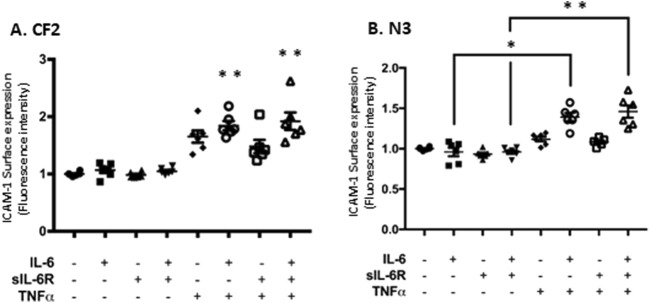
TNF-α and IL-6 co-stimulation increases ICAM-1 surface expression level in N3 and CF2 cell lines. Flow cytometry analysis of ICAM-1 surface expression level (geometric mean fluorescence intensity; GMFI) in CF2 **(A)** and N3 **(B)** cell lines after co-stimulation in absence or presence of IL-6, sIL-6Rα, and/or TNFα at 20 ng/mL for 24 h. Data are representative of six different experiments. The y-scale magnitude varies from one graph to the other. Data are presented as the mean ± SEM and were performed using the Kruskal–Wallis non-parametric test, followed by Dunn’s multiple comparisons post-test. Significance levels are shown as *(p < 0.05) and **(p < 0.001) compared to the untreated samples. N3, non-CF bronchial epithelial cells counterpart; CF2, CF bronchial epithelial cells; ICAM, inter-cellular adhesion molecule; TNF-α, tumor necrosis factor alpha; sIL-6Rα, soluble interleukin-6 receptor alpha; IL-6, interleukin-6.

The next step was to determine whether the increase in mRNA expression was translated into greater cell surface expression in N3 and CF2 using flow cytometry. BEAS-2B cells have higher basal expression of ICAM-1, which is likely explained by the nature of their immortalization, and thus, they were not included in this set of experiments. IL-6 classic and trans-signaling failed to increase ICAM-1 cell surface expression (Figure 6), but TNF-α alone modestly increased ICAM-1 surface expression in CF2 bronchial cells (Figure 6A). However, similarly to the previous figure, higher cell surface expression of ICAM-1 was observed when IL-6 classic and trans-signaling were combined with TNF-α, with the highest level achieved when TNF-α was combined with IL-6 trans-signaling in CF2.

Together, these results indicate that the combination of TNF-α and IL-6 trans-signaling has the greatest potential at increasing ICAM-1 in the CF2 cell line.

### 3.4 Interaction between bacterial proteases and host IL-6 signaling

**FIGURE 7:**
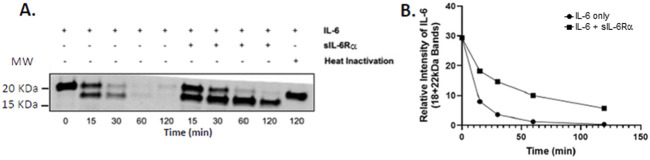
Presence of sIL-6Rα slows the rate of IL-6 degradation by *P. aeruginosa*-derived material. Filtered supernatants from planktonic *P. aeruginosa* (PA14 strain), prepared as described previously (Beaudoin et al., 2013), were incubated with IL-6 alone or in molar concentration matched with sIL-6Rα at 478 nM for the indicated times at 37 °C. Lysates were immunoblotted using the anti-IL-6 antibody **(A)**. Data are representative of three experiments, which were quantified by combining the signal intensity of the 18- and 22-kDa bands **(B)**. sIL-6Rα, soluble interleukin-6 receptor alpha; IL-6, interleukin-6.

Until now, the focus has been on host factors influencing IL-6 trans-signaling, and CF lungs are present in the majority of patients infected by *P. aeruginosa* (LaFayette et al., 2015; Ruffin et al., 2018; Hennemann et al., 2021). *P. aeruginosa*-derived factors can profoundly alter the host defense response (Faure et al., 2018). Proteases regulated by LasR, such as LasB from *P. aeruginosa*, efficiently degrade IL-6, thereby inactivating cytokines in the proximity of growing bacteria (LaFayette et al., 2015). However, the impact of sIL-6Rα on IL-6 degradation by *P. aeruginosa*-derived proteases is not known. To test this, we performed an *in vitro* IL-6 degradation assay using PsaDM filtrates derived from the PA14 virulent strain as the source of active proteases. “Naked” IL-6 was degraded more quickly (loss of the 18 and 22 kDa bands) than when equimolar sIL-6Rα was added (Figure 7). Degradation can also be prevented by inactivating PsaDM using heat, which destroys protease activity (Figure 7) (LaFayette et al., 2015). Therefore, protecting IL-6 from proteases is another mechanism by which trans-signaling could lead to a more sustained inflammatory state.

## 4 Discussion

In CF, persistent infiltration of neutrophils into the airways is the result of the presence of many pro-inflammatory cytokines, including IL-8, IL-6, and TNF-α (Cabrini et al., 2020; Castaldo et al., 2020; Bonfield et al., 1999), along with other molecules and bacterial products (Sagel et al., 2009). To further our understanding of the mechanisms that orchestrate the high inflammatory profile in the lungs of pwCF, we sought to characterize the role of IL-6 signaling in PEx and its contribution to inflammation.

Several studies have shown elevated IL-6 levels in various inflammatory diseases (Tanaka et al., 2014; Strand et al., 2020; Gabay, 2006). However, despite the evidence of persistent significant inflammatory activity, IL-6 expression levels remain low during PEx of pwCF (Osika et al., 1999; Colombo et al., 2005). *In vivo*, sIL-6Rα enhances IL-6 activity by 10–100 folds (Pet et al., 1996), resulting in strong responses to even very low doses of IL-6, such as those found in the CF lung. This is congruent with our prior observation that IL-6 levels in pwCF fall within the physiological range (5 pg/mL–15 pg/mL) in our cohort, whether at baseline (2.93 pg/mL) or during exacerbation (6.0 pg/mL) (Wojewodka et al., 2014). Our observation that CF2 cells lead to STAT3 phosphorylation at lower IL-6 concentrations suggests that they can trigger inflammatory signaling much earlier than N3 or BEAS-2B cells. In peripheral blood, soluble gp130 (sgp130) is a naturally occurring antagonist and has highlighted several important activities of the IL-6 trans-signaling pathway during acute inflammatory responses. Circulating levels of sgp130 are reported to be 400 ng/mL (Calabrese and Rose-John, 2014). Interestingly, we found that circulating levels of sgp130 averaged 250 ng/mL in patients experiencing exacerbation. It is unclear whether this lower value in our cohort reflects a physiologically relevant phenomenon. Nevertheless, both values are in large excess of the circulating sIL-6Rα we noted in patients experiencing exacerbation (∼30 ng/mL). The buffering capacity of the blood is rarely exceeded, except in some instances such as sepsis, whereas the circulating levels of IL-6 can reach the µg levels (Calabrese and Rose-John, 2014). In contrast, shedding of sIL-6Rα occurs at areas of active inflammation and where neutrophils are highly abundant, such as the inflamed airways of pwCF. Therefore, in the airways, sIL-6Rα can easily exceed the concentrations of sgp130 and engage trans-signaling with the membrane-bound gp130. This is in accordance with reports showing that sIL-6Rα acts mainly locally where it is generated (Peters et al., 1997).

A single-nucleotide polymorphism in the IL-6Rα (rs222815; IL-6R[D358A]) has been linked with a 35% increase in sIL-6Rα per 358A allele (Ferreira et al., 2013). This appears to be linked with greater shedding of the receptor. Interestingly, humans harboring the 358A allele have lower membrane IL-6Rα, leading to a decrease in classic signaling, whereas the increased abundance of sIL-6Rα favors trans-signaling (Ferreira et al., 2013). In the lungs, this phenotype has been associated with severe asthma and worse lung function (Hawkins et al., 2012). Considering that lower levels of sIL-6Rα are associated with better lung function, it leads us to speculate that IL-6 trans-signaling plays a pathologic role in CF lung diseases.

Concomitantly, leukocyte–endothelial adhesion molecules such as intercellular adhesion molecule-1 (ICAM-1) play a crucial role in the recruitment and regulation of neutrophil migration (Chong et al., 2021) and the inflammatory response (Mrugacz et al., 2007; Bui et al., 2020). It has been shown that neutrophils preferentially accumulate in the CF lung epithelium that overexpresses ICAM-1 compared to normal lung epithelium (Hubeau et al., 2001). Interestingly, our *in vitro* experimental models with CF2 showed that ICAM-1 mRNA levels were more strongly upregulated after stimulation with IL-6 and were particularly responsive to trans-signaling compared to non-CF bronchial cells. This suggests that ICAM-1 may provide a mechanism for retaining neutrophils at sites where they are needed and, thus, maintaining the pro-inflammatory state. Furthermore, we observed increased ICAM-1 expression during IL-6 signaling in conjunction with TNF-α signaling, suggesting a cooperative effect via the NF-κB and STAT-3 pathways and thereby promoting neutrophil recruitment. ICAM-1 is known to be upregulated by several cytokines, such as interferon-γ (IFN-γ), IL-1, IL-6, and IL-8 (Castaldo et al., 2020; Osika et al., 1999), and also by TNF-α via the NF-κB pathway (Copreni et al., 2009; Cohen-Cymberknoh et al., 2013), in addition to the IL-6-induced STAT-3 pathway (Rakemann et al., 1999; Sagwal et al., 2021; Zegeye et al., 2018). Whether this cooperative effect is also the result of gene transcription and increased mRNA stability driven by TNF-α is yet to be determined. ICAM-1, like many pro-inflammatory genes, is subject to post-transcriptional regulation via its 3′ untranslated mRNA region, which contains AU-rich elements. This represents an interesting avenue for further exploration. Interestingly, in N3 cells, while no differences were noted in mRNA expression in response to the co-stimulation (IL-6 + TNF-α), there was, nevertheless, an increase in protein expression that is congruent with post-transcriptional regulation. Additionally, CFTR-deficient airway epithelial cells impair glutathione transport, making the ADAM17 metalloprotease more active (Stolarczyk and Scholte, 2018) and resulting in ADAM17-mediated cleavage of the sIL-6R and activation of TNF-α (Gooz, 2010; Rose-John, 2017; Bell et al., 2007). This suggests that an interaction between the TNF-α and IL-6 trans-signaling pathways can occur and contribute to elevated inflammation in CF.

Neutrophils are not able to carry out complete bacterial clearance in the lungs of pwCF (Hayes et al., 2011; Laval et al., 2016), and the continued presence of *P. aeruginosa* leads to the secretion of proteases capable of degrading IL-6 (Endres et al., 2022). The gene encoding the major quorum-sensing transcriptional regulator in *P. aeruginosa*, *LasR*, is frequently inactivated, leading to increased levels of IL-6 cytokine via loss of the bacterial protease LasB, which can efficiently degrade it (LaFayette et al., 2015; Hennemann et al., 2021). This leads to greater inflammation and has been associated with pwCF who have the most severe cases of lung disease (LaFayette et al., 2015; Ng et al., 2020). Conversely, while “naked” IL-6 is susceptible to protease degradation (LaFayette et al., 2015), the presence of sIL-6Rα associated with trans-signaling protects IL-6 from degradation. Therefore, another mechanism by which trans-signaling can lead to a sustained inflammatory state, in contrast to classic signaling, is by maintaining IL-6 activity for longer.

We propose that at the onset of PEx, changes in microbial activity result in increased activation of AECs, leading to the synthesis of neutrophil chemoattractants (IL-8 and GROα) and IL-6. This first wave of inflammatory signaling leads to the recruitment of neutrophils and activates IL-6 classic signaling (low to moderate levels of STAT-3 activity). The newly recruited and activated neutrophils will, among other functions, shed their IL-6Rα to generate sIL-6Rα. Similarly, ADAM17 activity in stromal cells will further result in sIL-6Rα shedding. The net result of this second wave of inflammation will be decreased classic IL-6 signaling (due to the loss of the membrane receptor), which will favor IL-6 trans-signaling (high STAT-3 activity), leading to greater synthesis of pro-inflammatory factors. This will perpetuate inflammation for longer than required, resulting in excessive tissue damage. Antibiotic treatment given during PEx will finally removes the “insulting factors,” thus progressively leading to the termination of PEx, but not before excessive damage has already occurred.

## Data Availability

The raw data supporting the conclusions of this article will be made available by the authors, without undue reservation.

## Abbreviations

AECs, airway epithelial cells; CF, cystic fibrosis; pwCF, people with CF; IL, interleukin; PsaDM, *Pseudomonas aeruginosa* diffusible material; CFTR, cystic fibrosis transmembrane conductance regulator; STAT3, signal transducer and activator of transcription 3; TNF-α, tumor necrosis factor alpha; ICAM-1, intercellular adhesion molecule 1; ADAM17, ADAM metallopeptidase domain 17; PEx, pulmonary exacerbation.
